# Impact of Vascular Access Type and Obesity on Long-Term Thrombosis and Access Failure in Hemodialysis: A Real-World Cohort Study from the TriNetX Global Collaborative Network

**DOI:** 10.3390/biomedicines14061380

**Published:** 2026-06-18

**Authors:** Hung-Jin Huang, Pao-Ting Wu, Li-Chin Sung, Cai-Mei Zheng, Hui-Wen Chiu

**Affiliations:** 1 Graduate Institute of Clinical Medicine, College of Medicine, Taipei Medical University, Taipei 110301, Taiwan; 2 Division of Nephrology, Department of Internal Medicine, School of Medicine, College of Medicine, Taipei Medical University, Taipei 110301, Taiwan; 3 Division of Nephrology, Department of Internal Medicine, Shuang Ho Hospital, Taipei Medical University, New Taipei City 235041, Taiwan; 4 Division of Cardiology, Department of Internal Medicine, School of Medicine, College of Medicine, Taipei Medical University, Taipei 110301, Taiwan; 5 Division of Cardiology, Department of Internal Medicine, Shuang Ho Hospital, Taipei Medical University, New Taipei City 235041, Taiwan; 6 Taipei Heart Institute, Taipei Medical University, Taipei 110301, Taiwan; 7 Department of General Medicine, Shuang Ho Hospital, Taipei Medical University, New Taipei City 235041, Taiwan; 8 TMU Research Center of Urology and Kidney, Taipei Medical University, Taipei 110301, Taiwan; 9 Department of Medical Research, Shuang Ho Hospital, Taipei Medical University, New Taipei City 235041, Taiwan; 10 Ph.D. Program in Drug Discovery and Development Industry, College of Pharmacy, Taipei Medical University, Taipei 110301, Taiwan

**Keywords:** hemodialysis, arteriovenous fistula, arteriovenous graft, obesity, antithrombotic therapy

## Abstract

**Background/Objectives**: Optimal vascular access remains a critical determinant of outcomes in patients undergoing maintenance hemodialysis. While an arteriovenous fistula (AVF) is generally preferred over an arteriovenous graft (AVG), the impact of obesity and antithrombotic therapy on access-related complications remains incompletely defined. This study evaluated the association between vascular access type, obesity status, and adverse outcomes in a large real-world cohort. **Methods**: We conducted a retrospective cohort study using de-identified electronic health record data from the TriNetX Global Collaborative Network. Adult patients (≥18 years) receiving maintenance hemodialysis were stratified by vascular access type (AVF vs. AVG), body mass index (normal: 18.5–24.9 kg/m^2^, obese: ≥30 kg/m^2^), and antithrombotic medication exposure. Propensity score matching (1:1) was performed within BMI strata. Primary outcomes included vascular access thrombosis, AVG failure, and AVF failure. Time-to-event analyses used Kaplan–Meier and Cox proportional hazards models. **Results**: AVG was associated with significantly higher rates of thrombosis and access failure compared with AVF in both obese and normal-weight cohorts (all *p* < 0.0001). In patients with obesity, thrombosis rates increased from 10.47% (AVF) to 17.54% (AVG) at 3 months to 34.32% versus 42.24% at 5 years. Kaplan–Meier analysis demonstrated early and persistent separation of thrombosis-free survival curves, with AVG associated with increased risk (HR 1.23; 95% CI, 1.07–1.41; log-rank *p* = 0.0001). Antithrombotic therapy reduced absolute risks but did not eliminate the relative disadvantage of AVG. **Conclusions**: In this large real-world cohort, AVG was consistently associated with higher risks of thrombosis and access failure compared with AVF, regardless of obesity status or medication exposure. These findings support preferential use of AVF and highlight the need for individualized vascular access strategies in patients undergoing hemodialysis.

## 1. Introduction

End-stage kidney disease (ESKD) represents the terminal stage of chronic kidney disease, at which point renal replacement therapy becomes essential for survival [1]. Dialysis, including peritoneal dialysis (PD) and hemodialysis (HD), is a life-sustaining intervention for ESKD patients by removing metabolic waste, toxins, and excess fluid [2]. Due to its feasibility and convenience, HD remains the most widely utilized approach globally, and its effectiveness is critically dependent on the establishment and maintenance of reliable vascular access. Although tunneled central venous catheters may be used when immediate dialysis access is required, the arteriovenous fistula (AVF). Although tunneled central venous catheters may be used when immediate dialysis access is required, the arteriovenous fistula (AVF) [3] and the arteriovenous graft (AVG) [4] remain the two most commonly used permanent vascular access options for maintenance hemodialysis. Current KDOQI guidelines recommend AVF as the preferred vascular access whenever feasible because of its superior long-term patency and lower complication rates compared with AVG [5]. AVG involves placing an artificial blood vessel between an artery and a vein, using a synthetic, tube-like conduit to connect them, thus allowing earlier use, often within 2 to 3 weeks after implantation [6]. However, AVF is limited by a substantial risk of maturation failure and may require a prolonged time—typically 6 to 12 weeks—before it becomes suitable for repeated cannulation [7]. Despite increased infection risk, the AVG method is the only option when autologous vessels are unsuitable for AVF creation [8]. Thus, the choice between AVF and AVG is guided by multiple patient-specific factors [9], including vascular anatomy [10], comorbid conditions [11], life expectancy [12], and the anticipated timeline for dialysis initiation.

Obesity has emerged as a highly prevalent condition among patients with ESRD and poses a significant clinical challenge in dialysis management [13]. However, the impact of obesity on vascular access outcomes in hemodialysis remains uncertain. Although AVF is generally favored, obese patients often experience technical difficulties related to fistula creation, delayed maturation, and challenges in cannulation due to increased subcutaneous tissue thickness [14]. Therefore, AVG represents an alternative for patients when AVF creation is not feasible [15]. In contrast, graft-based access has significantly higher rates of thrombosis, stenosis, infection, and reduced access longevity. Patients often require repeated surgical or endovascular interventions to maintain access patency [16]. While obesity is a well-established risk factor for venous thromboembolic disease (VTE) in the general population [17], its specific influence on vascular-access-related complications and the comparative performance of AVF versus AVG remains not fully understood [18]. Most importantly, this uncertainty is further complicated by the frequent use of antithrombotic therapies in hemodialysis patients [19]. As a result, clinicians usually face uncertainty when choosing the optimal type of permanent vascular access between AVF and AVG in obese patients [20].

The TriNetX database is a global research network that aggregates de-identified electronic health record (EHR) data from healthcare organizations, biopharma companies, and contract research organizations (CROs) worldwide [21]. Moreover, TriNetX supports longitudinal data analysis, allowing researchers to track disease progression, intervention outcomes, and healthcare utilization over time [22]. Body mass index (BMI), as defined by the World Health Organization (WHO), was used to classify obesity status in this study [23]. It is calculated by dividing a person’s weight in kilograms by their height in meters squared (kg/m^2^). BMI serves as a useful population-level measure of overweight and obesity, as it is the same for both sexes and all age groups of adults. In this context, the present study leveraged a large, multi-institutional dataset to systematically compare vascular-access-related adverse outcomes between AVF and AVG in patients undergoing maintenance hemodialysis. Specifically, we evaluated thrombosis and access failure across predefined time points ranging from 3 months to 5 years, stratified by BMI category (normal weight vs. obesity) and medication exposure. Taken together, this study aimed to compare the risk of vascular access-related adverse outcomes between AVF and AVG in patients undergoing hemodialysis with prior vascular access failure, and to evaluate vascular-access-related outcomes stratified by obesity status and antithrombotic medication exposure.

## 2. Materials and Methods

### 2.1. Study Cohorts and Data Source

**Figure 1:**
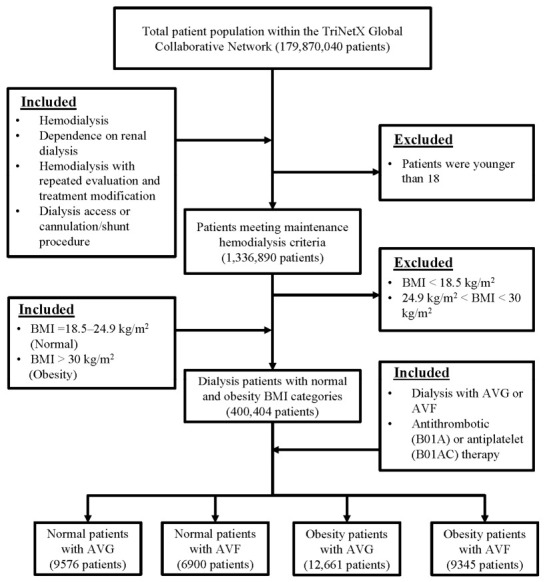
Flowchart of data selection for inclusion and exclusion in the adverse events study among dialysis patients.

This retrospective cohort analysis used anonymized patient data from the TriNetX Global Collaborative Network, a platform that aggregates real-time EHR data from a consortium of 143 healthcare institutions. Adult patients (aged ≥18 years) with maintenance hemodialysis were included using standardized diagnosis and procedure codes. Patients were classified according to permanent vascular access type as AVF or AVG. Body mass index (BMI) measurements recorded within 1 year before cohort entry were used to stratify participants into obese (BMI ≥ 30 kg/m^2^) and normal-weight (BMI 18.5–24.9 kg/m^2^) groups, according to WHO guidelines [24]. A total of 1,336,890 dialysis patients were initially screened from the TriNetX Global Collaborative Network. After application of predefined inclusion and exclusion criteria, 400,404 patients were eligible for the final analysis cohort. The detailed cohort selection process is summarized in Figure 1.

#### 2.1.1. Hemodialysis Population and Exclusion Criteria (Groups 1–2)

Maintenance hemodialysis status was identified using standardized diagnosis and procedure codes, including SNOMED CT code 302497006 (Hemodialysis), ICD-10-CM code Z99.2 (Dependence on renal dialysis), ICD-9-CM code 39.95 (Hemodialysis), and CPT codes 90937, 1006747, and 1012752. These criteria were used solely to identify the source population and exclude patients without maintenance hemodialysis for more than three months. BMI data were obtained from laboratory records (TriNetX laboratory code TNX:9083). BMI measurements recorded within 1 year prior to cohort entry were used to exclude patients who did not meet predefined BMI categories. Patients were excluded if BMI data were missing or fell outside the normal-weight (BMI 18.5–24.9 kg/m^2^) or obese (BMI ≥ 30 kg/m^2^) ranges. No outcome analyses were performed for Groups 1 or 2.

#### 2.1.2. Analytic Cohort Definition for Vascular Access Failures (Group 3)

The final analytic cohort (Group 3) consisted of patients undergoing maintenance hemodialysis who met all inclusion criteria and were classified according to permanent vascular access type as AVG or AVF. Vascular access failure was defined using ICD-10-CM codes T82.857A and T82.868A for AVG, and T82.510A and T82.590A for AVF. Only patients with documented vascular access failure events occurring on or before 1 January 2020, were included.

#### 2.1.3. Index Date and Landmark Design

To ensure appropriate temporal alignment between exposure and outcomes and to minimize immortal-time bias, a 3-month landmark design was applied. The index date was defined as the first documented AVF or AVG placement following initiation of maintenance hemodialysis. Patients were categorized by the vascular access type present on the index date. To minimize immortal-time bias and ensure adequate vascular access maturation, a 90-day landmark period was applied. Only patients who survived and remained under follow-up during this period were included. Outcome assessment began on Day 91 after the index date.

### 2.2. Data Analysis and Covariate Balance Assessment

Study outcomes were defined according to the standardized outcome definitions implemented within the TriNetX platform. The evaluated outcomes included vascular-access-related thrombosis, CVA, CHF, infection, peripheral artery disease (PAD), acute myocardial infarction (AMI), AVG failure, and AVF failure. Thrombosis was defined using the ICD-10-CM code T82.898A, which represents other specified complications of vascular prosthetic devices, implants, and grafts. CVA was identified using ICD-10-CM codes I60–I69; CHF using ICD-10-CM code I50; PAD using ICD-10-CM code I70.2; and AMI using ICD-10-CM code I21. Infection was defined as inflammation and infection associated with cardiac and vascular devices, implants, and grafts (ICD-10-CM: T82.7XXA). AVG failure was defined using ICD-10-CM codes T82.857A (stenosis of vascular prosthetic devices) and T82.868A (thrombosis due to vascular prosthetic devices). In contrast, AVF failure was defined using ICD-10-CM codes T82.590A (other mechanical complication of surgically created arteriovenous fistula) and T82.510A (breakdown of surgically created arteriovenous fistula). These outcome definitions were selected to capture clinically relevant vascular-access-related complications using standardized diagnostic codes available within the TriNetX database and were applied uniformly across all study cohorts. To our knowledge, formal validation studies specifically evaluating these coding algorithms for AVF- and AVG-related outcomes within the TriNetX platform remain limited. All outcomes were evaluated using risk analysis and Kaplan–Meier survival analysis, as supported by the TriNetX platform. The abovementioned outcomes were assessed at predefined follow-up intervals of 3 months, 6 months, 1 year, 3 years, and 5 years following the landmark index date.

### 2.3. Propensity Score Matching

To minimize confounding and enhance comparability between the AVF and AVG cohorts, 1:1 propensity score matching was performed separately within obese and normal-BMI strata using the TriNetX platform. The propensity score model incorporated baseline laboratory profiles (Table S1), demographic characteristics (Table S2), and clinical diagnoses and concomitant medication classes (Table S3). Laboratory covariates included electrolytes and renal/metabolic markers (e.g., potassium, sodium, calcium, phosphate, glucose, creatinine, and urea nitrogen), hematologic indices (hemoglobin and hematocrit), nutritional markers (albumin and total protein), liver enzymes (AST, ALT, and alkaline phosphatase), lipid parameters (total cholesterol, triglycerides, HDL, and LDL), endocrine/cardiac biomarkers (intact parathyroid hormone, BNP, NT-proBNP), and calcidiol. Demographic variables included sex and race categories. Clinical covariates included significant comorbidities (e.g., hypertensive diseases, diabetes mellitus, ischemic heart disease, and cerebrovascular disease) as well as commonly used cardiovascular and metabolic medication classes (e.g., antiarrhythmics, beta blockers, glucose-lowering agents, antilipemic agents, calcium channel blockers, diuretics, ACE inhibitors, angiotensin II inhibitors, and alpha blockers). Covariate balance before and after matching was assessed using standardized mean differences (SMDs), with an absolute SMD < 0.10 prespecified as adequate balance. Following matching, the AVF and AVG cohorts achieved improved balance across laboratory, demographic, and clinical domains in both BMI strata, with SMDs reduced to below the prespecified threshold as summarized in Tables S1–S3, supporting the comparability of the matched cohorts for subsequent outcome analyses.

### 2.4. Statistical Analysis

All statistical analyses were performed using the TriNetX platform. Propensity score matching was conducted at a 1:1 ratio using greedy nearest-neighbor matching to balance baseline characteristics between AVF and AVG cohorts. Time-to-event outcomes were analyzed using the Kaplan–Meier method, with between-group comparisons assessed using the log-rank test. Cox proportional hazards models were used to estimate hazard ratios (HR) with 95% confidence intervals. Patients with outcome events occurring before the start of the analysis window were excluded from the corresponding analyses. A two-sided *p*-value < 0.05 was considered statistically significant.

## 3. Results

### 3.1. Baseline Characteristics

The final matched cohorts included Normal AVF (*n* = 6900), Normal AVG (*n* = 9576), Obesity AVF (*n* = 9345), and Obesity AVG (*n* = 12,661) patients. During the 3-month to 5-year follow-up period, the AVG group exhibited significantly higher risks of thrombosis and vascular access failure compared with the AVF group. Among obesity patients, those with and without medication both demonstrated elevated ORs for adverse outcomes following AVG creation, with temporal variations across observation periods. Similarly, in patients with normal BMI, the forest plots for AVF creation with and without medication showed smaller but still notable differences in adverse event risk. Overall, the analysis showed that obesity and the use of certain medications were associated with higher odds of complications, particularly within the first year after vascular access creation. Across the 3-month to 5-year follow-up period, the AVG group had a significantly higher risk of thrombosis than the AVF group (HR = 1.23). The curves diverged early during follow-up and remained separated throughout the observation window, highlighting the long-term impact of access type on vascular patency. Among obese patients, thrombosis, AVG failure, and AVF failure increased progressively over time in both cohorts. Across all follow-up intervals, the AVG group consistently demonstrated higher cumulative incidences of adverse vascular access outcomes than the AVF group, regardless of medication exposure. Similar patterns were observed among normal-weight patients, indicating that the associations between vascular access type and adverse outcomes remained consistent across BMI categories and medication exposure groups (Table 1).

Comparable trends were observed in normal-weight patients. In the medication group, thrombosis rates increased from 10.16% to 16.18% at 3 months and from 28.40% to 38.54% at 5 years in the AVF group and the AVG group, respectively. AVG and AVF failure rates followed parallel trajectories, with the AVG group consistently demonstrating significantly higher cumulative incidences across all follow-up intervals (*p* < 0.0001). Notably, similar patterns persisted in the no-medication subgroup, indicating that the observed differences between cohorts were robust across medication exposure strata. Overall, these findings demonstrate a time-dependent increase in adverse vascular access outcomes, with the AVG group showing persistently higher risks than the AVF group, independent of obesity status and medication use.

### 3.2. Covariate Balance After Matching

Baseline characteristics of patients receiving AVG or AVF, stratified by obesity status, are summarized in Tables S1–S3. Before propensity score matching, baseline laboratory (Table S1), demographic (Table S2), and clinical characteristics, including comorbidities and medication use (Table S3), were generally comparable between groups. However, modest imbalances were observed for selected variables, with several SMDs approaching or exceeding 0.10. Following 1:1 propensity score matching, substantial balance was achieved across all domains. Post-matching comparisons demonstrated highly similar distributions of laboratory values, demographic characteristics, clinical diagnoses, and concomitant medication use between the AVG and AVF groups, with all SMDs < 0.10 in both the obese and non-obese populations. These findings indicate that propensity score matching effectively minimized baseline imbalances and controlled for potential confounding between cohorts.

### 3.3. Measures of Association of Obesity and Normal Patients

The cumulative incidence of thrombosis, AVG failure, and AVF failure from 3 months to 5 years was evaluated among obese and normal-weight patients, stratified by medication exposure and cohort classification, with detailed estimates summarized in Table 1. Across all follow-up intervals and outcome measures, the AVF group consistently demonstrated significantly lower event rates than the AVG group, with all between-group comparisons reaching strong statistical significance (*p* < 0.0001).

Among obese patients receiving medication, the incidence of thrombosis increased progressively over time in both cohorts. Still, it remained consistently lower in the AVF group than in the AVG group, rising from 10.47% versus 17.54% at 3 months to 34.32% versus 42.24% at 5 years. AVG failure exhibited a substantially higher cumulative incidence than thrombosis, with rates increasing from 36.03% versus 21.97% at 3 months to 58.42% versus 45.19% at 5 years in the AVF group and the AVG group, respectively. Similarly, AVF failure rates increased steadily over the follow-up period, with the AVF group maintaining a markedly lower incidence than the AVG group at all time points. In obese patients without medication exposure, comparable trends were observed. Thrombosis, AVG failure, and AVF failure rates were uniformly higher in the AVG group than in the AVF group across all follow-up intervals, and the absolute differences between cohorts became more pronounced at longer durations, particularly at 3 and 5 years. Normal-weight patients had a lower cumulative incidence of all outcomes than obese patients. In the medication group, thrombosis rates increased from 10.29% in the AVF group and 16.02% in the AVG group at 3 months to 29.89% and 37.46%, respectively, at 5 years. AVG failure remained the most frequent adverse outcome, accounting for 54.62% in the AVF group and 42.46% in the AVG group at the 5-year follow-up. In contrast, AVF failure showed a similar pattern of progressive increase with consistently lower rates in the AVF group. In the AVF group, patients without medication, the normal-weight patients continued to exhibit significantly reduced risks of thrombosis, AVG failure, and AVF failure compared with the AVG group throughout the observation period, with a persistent separation between cohorts from early to late follow-up.

### 3.4. Forest Plots of AVG and AVF

**Figure 2:**
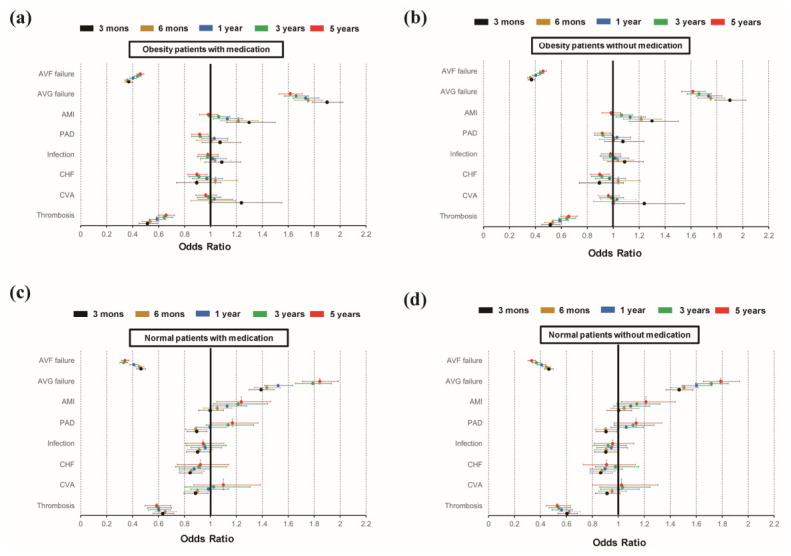
Forest plot of obesity patients for arteriovenous graft (AVG) creation with (**a**) or without (**b**) medication. Forest plot of normal BMI patients for arteriovenous fistula (AVF) creation with (**c**) or without (**d**) medication. The data show the odds ratios for adverse events across different observation times.

The forest plots illustrating the associations between medication use and the risk of adverse events across different observation periods, stratified by BMI category and type of vascular access, are shown in Figure 2. Among obese patients undergoing AVG creation, medication use was consistently associated with lower odds of adverse events compared with non-users across all follow-up intervals (Figure 2a), whereas obese patients without medication exposure demonstrated comparatively higher and more variable risk estimates (Figure 2b). In contrast, among patients with normal BMI undergoing AVF creation, medication use was similarly associated with reduced adverse event risk across observation periods (Figure 2c), whereas non-medicated patients with normal BMI exhibited attenuated or neutral associations (Figure 2d). Overall, these findings indicate that the protective association of medication use was observed in both BMI-defined subgroups, with a more pronounced and consistent effect among obese patients receiving AVG creation. “Collectively, the subgroup analyses suggest that differences in adverse clinical outcomes were observed across BMI and vascular access subgroups following access creation.”

### 3.5. Time-Dependent Differences in Thrombosis-Free Survival Between AVF and AVG

**Figure 3:**
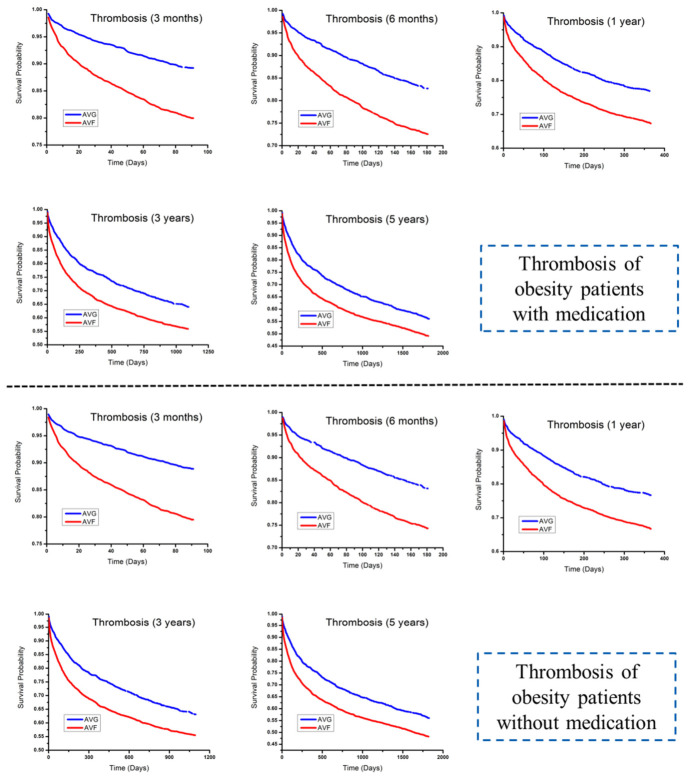
Kaplan–Meier survival curves of obesity patients for the 3-month to 5-year association of thrombosis with or without medication. The data comparing adverse events between the AVG and AVF groups: The AVG group had a significantly higher risk than the AVF group (HR = 1.23, 95% CI: 1.07–1.41; log-rank *p* = 0.0001).

**Figure 4:**
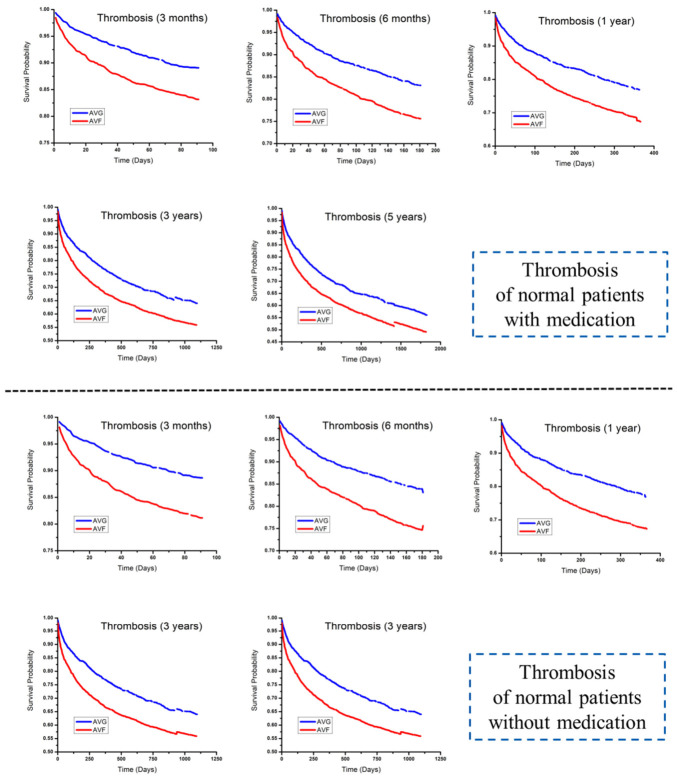
Kaplan–Meier survival curves of normal patients for the 3-month to 5-year association of thrombosis with or without medication. The data comparing adverse events between the AVG and AVF groups indicates a significantly increased risk in the AVG group compared to the AVF group (log-rank *p* < 0.05).

Kaplan–Meier survival analyses were performed to compare time-to-event outcomes between AVF and AVG at 3-, 6-, 1-, 3-, and 5-year follow-up intervals (Figure 3 and Figure 4 and Figures S1–S4). For thrombosis, the thrombosis-free survival curves demonstrated clear and persistent separation between access types over time. In obese patients, the AVG group exhibited a more rapid decline in thrombosis-free survival than the AVF group, with the curves diverging during the early follow-up period (3–6 months) and remaining divergent throughout intermediate- and long-term follow-up (Figure 3). Log-rank testing confirmed a statistically significant difference in thrombosis-free survival between AVG and AVF across the entire follow-up period. Similar time-dependent patterns were observed among normal-weight patients. Kaplan–Meier curves showed consistently lower thrombosis-free survival in the AVG group than in the AVF group, with early separation of the curves and sustained differences over extended follow-up (Figure 4). These findings indicate that the elevated thrombosis risk associated with AVG relative to AVF is present from early after access creation and persists over time, irrespective of BMI category. Time-to-event analyses for access failure further demonstrated marked differences between access types. For both obese and normal-weight patients, the survival curves for access failure outcomes revealed consistently lower event-free survival probabilities in the AVG group compared with the AVF group. Curve separation was observed early after access creation and widened progressively with increasing follow-up duration, reflecting cumulative differences in access durability between prosthetic grafts and autogenous fistulas (Figures S1–S4). The supplementary analyses showed concordant patterns across medication and BMI strata, supporting the robustness of the observed time-dependent differences.

## 4. Discussion

In this large real-world cohort of patients undergoing hemodialysis, we observed consistent, time-dependent differences in vascular access-related adverse outcomes across cohorts defined by access type, BMI, and medication exposure. The present study focused on comparing normal-weight and obese patients. Therefore, individuals with a BMI of 25–29.9 kg/m^2^ were not included in the predefined study design. Future studies are warranted to evaluate vascular access outcomes in this clinically relevant subgroup. Across follow-up intervals from 3 months to 5 years, the AVG group demonstrated significantly higher cumulative incidence of thrombosis and AVG failure than the AVF group, with these differences remaining significant after propensity score matching. A central observation of this study is the progressive, cumulative nature of adverse events over time, particularly thrombosis and access failure. The time-to-event analyses demonstrated early divergence of thrombosis-free survival curves between AVF and AVG, with separation evident within the first 3 to 6 months following access creation and persisting throughout long-term follow-up. These observations suggest that additional risks associated with transplanted vascular access are not limited to the near-surgical period but persist and accumulate with long-term use. In contrast, AVF exhibits more stable event-free survival over time, consistent with its established status as the preferred long-term vascular access method.

Thrombosis appeared more strongly associated with AVG than AVF across the entire follow-up period. Thrombosis incidence was consistently higher in the AVG group than in the AVF group from 3 months to 5 years (Table 1), and Kaplan–Meier curves showed early and persistent separation of thrombosis-free survival (Figure 3 and Figure 4). These patterns suggest that graft-based access is inherently and persistently thrombotic, potentially related to the prosthetic surface and flow disturbances at the graft-vein anastomosis that predispose to stenosis and subsequent thrombosis. After adjusting for medication exposure, the association between thrombosis and graft-based access remained evident despite antithrombotic therapy. Based on antithrombotic medication exposure, including agents classified under ATC codes B01A and B01AC, thrombosis incidence was consistently higher in the AVG cohort than in the AVF cohort across all follow-up periods from 3 months to 5 years. Although patients receiving antithrombotic therapy demonstrated lower absolute event rates than those without medication, the relative disadvantage associated with AVG persisted with or without medication (Figure 2). The persistent separation of thrombosis-free survival curves between medicated and non-medicated groups indicates that the mechanisms driving thrombosis in AVG are not solely mediated by platelet activation pathways targeted by B01A/B01AC agents. However, a major limitation of this study is the potential for selection bias related to vascular access choice. In clinical practice, AVGs are often selected for patients with poor vascular anatomy or higher comorbidity burden, which may inherently predispose them to worse outcomes. Although propensity score matching was applied to balance observed characteristics, residual confounding due to unmeasured factors, and particularly vascular suitability, cannot be fully excluded. In addition, competing risks such as death were not explicitly modeled in this study. Given the high mortality rate among dialysis patients, this may have influenced the estimated associations derived from Kaplan–Meier and Cox analyses.

These findings are also supported by the established pathophysiology of hemodialysis graft failure [25]. Previous studies have shown that AVG thrombosis is most commonly a consequence of progressive stenosis, primarily driven by neointimal hyperplasia and smooth muscle proliferation, which reduces access flow and precipitates thrombotic occlusion [26]. These processes are strongly influenced by local hemodynamics (e.g., turbulence and abnormal shear stress at the junction between the graft and the vein) and by remodeling responses within the outflow vein, providing a mechanistic basis for the persistence of thrombosis risk despite systemic antithrombotic therapy [27]. In addition, computational simulations further confirmed that the geometric features of AVG may influence local flow, with high wall shear stress at the graft-vein anastomosis exacerbating flow disturbances that promote unsuitable venous remodeling [28]. Moreover, structural remodeling and stenosis-driven thrombosis in AVG suggest that reliance on systemic antithrombotic agents (ATC B01A/B01AC) may be insufficient to mitigate thrombotic risk fully [26]. However, these agents primarily affect platelet aggregation rather than local vascular wall changes. In addition, medication exposure was modeled as a baseline variable and did not account for time-varying changes during follow-up. In clinical practice, antithrombotic therapy is dynamic and may be initiated, discontinued, or dose-adjusted over time [29]. Failure to account for these temporal changes may introduce exposure misclassification and bias the estimated associations. In addition, a double-blind trial evaluating combined aspirin and clopidogrel therapy demonstrated no significant reduction in AVG thrombosis in hemodialysis patients [30], suggesting that graft failure is driven predominantly by access-specific structural and hemodynamic mechanisms rather than platelet activation alone. Thus, it cannot be fully mitigated by intensified antiplatelet therapy. Notably, although obesity was associated with a higher burden of vascular-access-related complications in the present study, previous studies have described an obesity paradox in hemodialysis populations, whereby overweight and moderately obese patients may demonstrate improved survival outcomes [31]. Therefore, the vascular-access-related findings observed in this study should not be extrapolated to overall survival outcomes.

Several limitations of this study should be acknowledged. First, because this investigation was based on a retrospective observational design using the TriNetX Global Collaborative Network, the findings remain susceptible to residual confounding despite extensive propensity score matching. Detailed information regarding vascular anatomy, vessel caliber, surgeon expertise, and access suitability was not uniformly available and therefore could not be fully adjusted for in the analysis. Second, important vascular access-specific factors, including AVF/AVG location (e.g., forearm, upper arm, or femoral access), access blood flow characteristics, cannulation techniques (such as stepladder, buttonhole, or area puncture methods), and dialysis-unit practice patterns, were not available within the TriNetX database and therefore could not be incorporated into the matching process. These factors may independently influence thrombosis and access failure rates, representing potential sources of residual confounding. In addition, patients selected for AVG placement may differ substantially from those receiving AVF with respect to vascular anatomy, vessel quality, fistula maturation potential, and overall vascular disease burden. Consequently, residual confounding and confounding by indication cannot be completely excluded. The observed associations between vascular access type and adverse outcomes should not be interpreted as causal relationships. Patients selected for AVG placement may have had more complex vascular disease, poorer vessel quality, or greater comorbidity burden, which could independently contribute to worse outcomes. Third, medication exposure was evaluated primarily at baseline and did not account for temporal changes in antithrombotic therapy, including treatment initiation, discontinuation, adherence, or dose adjustment during follow-up. Furthermore, information regarding treatment indication, cumulative exposure, and time-varying medication use was not available. Therefore, the observed medication-associated findings should be interpreted cautiously, as they may be affected by exposure misclassification and residual confounding during long-term follow-up.

Obesity was associated with a higher absolute burden of thrombosis and access failure; however, the database lacked detailed anthropometric and body composition parameters that may further influence vascular access performance. Competing risks such as all-cause mortality and kidney transplantation were not explicitly modeled and may have influenced long-term event estimates. Given the high mortality burden among patients receiving maintenance hemodialysis, standard Kaplan–Meier analyses may overestimate cumulative event probabilities when competing events are not considered. Therefore, the reported incidence estimates should be interpreted with caution, and future studies incorporating competing-risk methodologies are warranted. Furthermore, important factors related to vascular access selection and performance, including access location (e.g., forearm, upper arm, or femoral), vascular anatomy, vessel quality, surgeon preference, cannulation techniques, and dialysis-unit practice patterns, were not available in the TriNetX database. These unmeasured variables may have influenced both the choice between AVF and AVG and subsequent vascular access outcomes, resulting in residual confounding and potential confounding by indication. Therefore, we observed that associations should be interpreted as observational findings rather than evidence of a causal relationship between vascular access type and clinical outcomes. Despite these limitations, the large multicenter cohort and extended longitudinal follow-up provide important real-world evidence regarding the comparative performance of AVF and AVG in obese and non-obese hemodialysis populations. Furthermore, although analyses were stratified according to obesity status, a formal interaction analysis between obesity and vascular access type was not performed. Thus, differences observed between obese and normal-weight patients should be interpreted as descriptive subgroup findings rather than evidence of statistically confirmed effect modification.

## 5. Conclusions

In this large real-world cohort analysis, vascular access type was a major determinant of long-term adverse outcomes in patients undergoing hemodialysis. Compared with AVG, AVF was consistently associated with lower risks of thrombosis and superior long-term durability across all BMI categories and medication strata. Among the BMI categories evaluated in this study, obese patients demonstrated a higher absolute burden of vascular access-related adverse events than normal-weight patients. These findings support an individualized approach to vascular access selection, prioritizing AVF whenever anatomically feasible, particularly in patients with elevated BMI. Pharmacologic therapy should be regarded as an adjunctive strategy rather than a substitute for optimal access selection. Future prospective investigations incorporating advanced vascular imaging, mechanistic biomarkers, and time-dependent treatment modeling are warranted to refine risk stratification and optimize vascular access outcomes in the hemodialysis population. These findings apply specifically to the normal-weight and obese populations evaluated in this study and should not be generalized to all BMI categories.

## Figures and Tables

**Table 1:** Measures of association of obesity and normal patients for the 3-month to 5-year association of significant outcomes with or without medication.

Obese Patients	Thrombosis			AVG Failure			AVF Failure		
Medication group	AVG group	AVF group	*p* value	AVG group	AVF group	*p* value	AVG group	AVF group	*p* value
3 months	10.471%	17.536%	<0.0001	36.028%	21.973%	<0.0001	12.559%	29.517%	<0.0001
6 months	16.119%	23.632%	<0.0001	42.89%	28.197%	<0.0001	17.125%	35.596%	<0.0001
1 year	21.687%	29.784%	<0.0001	49.079%	34.102%	<0.0001	22.124%	41.343%	<0.0001
3 years	30.446%	38.205%	<0.0001	56.089%	42.027%	<0.0001	29.418%	48.921%	<0.0001
5 years	34.321%	42.236%	<0.0001	58.415%	45.185%	<0.0001	32.765%	52.152%	<0.0001
No medication group	AVG group	AVF group	*p* value	AVG group	AVF group	*p* value	AVG group	AVF group	*p* value
3 months	10.583%	17.898%	<0.0001	36.36%	22.168%	<0.0001	12.578%	30.154%	<0.0001
6 months	15.686%	24.067%	<0.0001	43.254%	28.191%	<0.0001	17.757%	36.087%	<0.0001
1 year	21.119%	30.151%	<0.0001	49.36%	34.141%	<0.0001	22.48%	41.85%	<0.0001
3 years	29.851%	38.585%	<0.0001	56.34%	42.151%	<0.0001	29.679%	49.433%	<0.0001
5 years	33.441%	42.565%	<0.0001	58.66%	45.314%	<0.0001	33.184%	52.585%	<0.0001
**Normal Patients**	**Thrombosis**			**AVG Failure**			**AVF Failure**		
Medication group	AVG group	AVF group	*p* value	AVG group	AVF group	*p* value	AVG group	AVF group	*p* value
3 months	10.293%	16.019%	<0.0001	34.115%	21.043%	<0.0001	11.055%	28.703%	<0.0001
6 months	14.769%	20.893%	<0.0001	40.741%	26.603%	<0.0001	14.975%	33.295%	<0.0001
1 year	19.642%	26.449%	<0.0001	46.055%	32.393%	<0.0001	19.305%	38.839%	<0.0001
3 years	27.648%	34.288%	<0.0001	52.747%	39.806%	<0.0001	25.767%	45.383%	<0.0001
5 years	29.886%	37.457%	<0.0001	54.617%	42.464%	<0.0001	28.489%	48.024%	<0.0001
No medication group	AVG group	AVF group	*p* value	AVG group	AVF group	*p* value	AVG group	AVF group	*p* value
3 months	10.119%	16.722%	<0.0001	34.84%	21.39%	<0.0001	11.214%	29.331%	<0.0001
6 months	14.435%	21.537%	<0.0001	41.371%	26.916%	<0.0001	14.989%	33.819%	<0.0001
1 year	19.296%	27.012%	<0.0001	47.043%	32.556%	<0.0001	19.203%	39.216%	<0.0001
3 years	27.133%	34.697%	<0.0001	53.622%	39.929%	<0.0001	25.717%	45.908%	<0.0001
5 years	29.415%	37.863%	<0.0001	55.599%	42.619%	<0.0001	28.634%	48.452%	<0.0001

## Data Availability

Data were obtained through the TriNetX global federated health research network, following institutional approval and adherence to data-sharing agreements.

## Supplementary Materials

The following supporting information can be downloaded at: https://www.mdpi.com/article/10.3390/biomedicines14061380/s1. Figure S1: Kaplan–Meier survival curves of obesity patients for the 3-month to 5-year association of AVG with or without medication; Figure S2: Kaplan–Meier survival curves of obesity patients for the 3-month to 5-year association of AVF with or without medication; Figure S3: Kaplan–Meier survival curves of normal patients for the 3-month to 5-year association of AVG with or without medication; Figure S4: Kaplan–Meier survival curves of normal patients for the 3-month to 5-year association of AVF with or without medication; Table S1: Baseline labs characteristics of arteriovenous graft (AVG) and arteriovenous fistula (AVF) groups before and after propensity score matching; Table S2: Baseline demographic characteristics of AVG and AVF groups before and after propensity score matching; Table S3: Baseline diagnostic characteristics of AVG and AVF groups before and after propensity score matching.

## Abbreviations

The following abbreviations are used in this manuscript:

AMI	Acute myocardial infarction
AST	Aspartate aminotransferase
ALT	Alanine aminotransferase
ATC	Anatomical Therapeutic Chemical
AVG	Arteriovenous graft
AVF	Arteriovenous fistula
BMI	Body mass index
BNP	B-type natriuretic peptide
CI	Confidence interval
CPT	Current Procedural Terminology
CRO	Contract research organization
CVA	Cerebrovascular accident
EHR	Electronic health record
ESKD	End-stage kidney disease
ESRD	End-stage renal disease
HD	Hemodialysis
HDL	High-density lipoprotein
HR	Hazard ratio
ICD	International Classification of Diseases
LDL	Low-density lipoprotein
NT-proBNP	N-terminal pro-B-type natriuretic peptide
PAD	Peripheral artery disease
PD	Peritoneal dialysis
SMD	Standardized mean difference
SNOMED CT	Systematized Nomenclature of Medicine Clinical Terms
VTE	Venous thromboembolism
WHO	World Health Organization
