# Supplementary material for: Impact of Vascular Access Type and Obesity on Long-Term Thrombosis and Access Failure in Hemodialysis: A Real-World Cohort Study from the TriNetX Global Collaborative Network
Source: Biomedicines. 2026 Jun 18;14(6):1380. doi: 10.3390/biomedicines14061380 (PMC13296801; doi:10.3390/biomedicines14061380)
Supplement: Supplementary file 1 [file biomedicines-14-01380-s001.zip › biomedicines-4349370-supplementary.pdf]

Table S1. Baseline labs characteristics of arteriovenous graft (AVG) and arteriovenous fistula (AVF) groups before and after propensity score matching.

**Obese patients**	**Before Matching**			**After Matching**		
Characteristic Name: Labs	AVG	AVF	Std. Diff.	AVG	AVF	Std. Diff.
Potassium [Moles/volume] in Serum, Plasma or Blood	0.76	0.78	0.03	0.77	0.77	0.03
Glucose [Mass/volume] in Serum, Plasma or Blood	0.76	0.76	0.01	0.76	0.77	0.00
Creatinine [Mass/volume] in Serum, Plasma or Blood	0.74	0.75	0.08	0.75	0.75	0.08
Urea nitrogen [Mass/volume] in Serum, Plasma or Blood	0.74	0.75	0.01	0.75	0.75	0.01
Hematocrit [Volume Fraction] of Blood	0.74	0.74	0.03	0.74	0.74	0.03
Sodium [Moles/volume] in Serum, Plasma or Blood	0.74	0.74	0.02	0.74	0.74	0.01
Calcium [Mass/volume] in Serum, Plasma or Blood	0.73	0.73	0.01	0.74	0.74	0.02
Hemoglobin [Mass/volume] in Blood	0.72	0.73	0.01	0.73	0.73	0.01
Albumin [Mass/volume] in Serum, Plasma or Blood	0.60	0.59	0.01	0.59	0.59	0.02
Aspartate aminotransferase [Enzymatic activity/volume] in Serum or Plasma	0.57	0.56	0.02	0.56	0.56	0.01
Alkaline phosphatase [Enzymatic activity/volume] in Serum, Plasma or Blood	0.57	0.56	0.02	0.56	0.56	0.02
Alanine aminotransferase [Enzymatic activity/volume] in Serum, Plasma or Blood	0.56	0.56	0.02	0.56	0.56	0.01
Phosphate [Mass/volume] in Serum, Plasma or Blood	0.54	0.54	0.07	0.54	0.54	0.07
Protein [Mass/volume] in Serum or Plasma	0.53	0.53	0.06	0.53	0.53	0.05
Hemoglobin A1c/Hemoglobin.total in Blood	0.36	0.36	0.01	0.36	0.36	0.01
Cholesterol [Mass/volume] in Serum or Plasma	0.25	0.24	0.06	0.24	0.24	0.03
Triglyceride [Mass/volume] in Serum, Plasma or Blood	0.25	0.24	0.01	0.24	0.24	0.01
Cholesterol in HDL [Mass/volume] in Serum or Plasma	0.24	0.23	0.02	0.23	0.23	0.01
Cholesterol in LDL [Mass/volume] in Serum or Plasma	0.24	0.23	0.04	0.23	0.23	0.01

Parathyrin.intact [Mass/volume] in Serum or Plasma	0.25	0.22	0.03	0.23	0.22	0.02
Natriuretic peptide B [Mass/volume] in Serum, Plasma or Blood	0.16	0.16	0.07	0.16	0.16	0.03
Calcidiol [Mass/volume] in Serum or Plasma	0.09	0.07	0.01	0.07	0.07	0.01
Natriuretic peptide.B prohormone N-Terminal [Mass/volume] in Serum, Plasma or Blood	0.06	0.06	0.13	0.06	0.06	0.09

Table S1. Continued.

Normal patients	Before Matching			After Matching		
Characteristic Name: Labs	AVG	AVF	Std. Diff.	AVG	AVF	Std. Diff.
Potassium [Moles/volume] in Serum, Plasma or Blood	0.79	0.80	0.06	0.78	0.79	0.05
Glucose [Mass/volume] in Serum, Plasma or Blood	0.78	0.78	0.02	0.77	0.78	0.01
Creatinine [Mass/volume] in Serum, Plasma or Blood	0.76	0.77	0.01	0.76	0.77	0.01
Urea nitrogen [Mass/volume] in Serum, Plasma or Blood	0.76	0.77	0.00	0.76	0.77	0.00
Hematocrit [Volume Fraction] of Blood	0.77	0.77	0.10	0.76	0.77	0.08
Sodium [Moles/volume] in Serum, Plasma or Blood	0.76	0.76	0.03	0.76	0.76	0.02
Calcium [Mass/volume] in Serum, Plasma or Blood	0.76	0.76	0.03	0.75	0.76	0.02
Hemoglobin [Mass/volume] in Blood	0.75	0.75	0.00	0.74	0.75	0.00
Albumin [Mass/volume] in Serum, Plasma or Blood	0.64	0.63	0.02	0.63	0.63	0.05
Aspartate aminotransferase [Enzymatic activity/volume] in Serum or Plasma	0.62	0.62	0.02	0.61	0.62	0.01
Alkaline phosphatase [Enzymatic activity/volume] in Serum, Plasma or Blood	0.62	0.62	0.02	0.61	0.62	0.03
Alanine aminotransferase [Enzymatic activity/volume] in Serum, Plasma or Blood	0.62	0.61	0.01	0.61	0.61	0.01
Phosphate [Mass/volume] in Serum, Plasma or Blood	0.59	0.57	0.06	0.57	0.58	0.06
Protein [Mass/volume] in Serum or Plasma	0.58	0.57	0.09	0.57	0.58	0.09
Hemoglobin A1c/Hemoglobin.total in Blood	0.31	0.30	0.00	0.29	0.30	0.00
Cholesterol [Mass/volume] in Serum or Plasma	0.25	0.24	0.01	0.24	0.24	0.01
Triglyceride [Mass/volume] in Serum, Plasma or Blood	0.26	0.23	0.06	0.23	0.23	0.03
Cholesterol in HDL [Mass/volume] in Serum or Plasma	0.24	0.23	0.02	0.23	0.23	0.02
Cholesterol in LDL [Mass/volume] in Serum or Plasma	0.24	0.22	0.09	0.22	0.22	0.05

Parathyrin.intact [Mass/volume] in Serum or Plasma	0.24	0.22	0.06	0.22	0.22	0.06
Natriuretic peptide B [Mass/volume] in Serum, Plasma or Blood	0.17	0.17	0.04	0.16	0.17	0.01
Calcidiol [Mass/volume] in Serum or Plasma	0.09	0.08	0.01	0.08	0.08	0.00
Natriuretic peptide.B prohormone N-Terminal [Mass/volume] in Serum, Plasma or Blood	0.04	0.05	0.06	0.05	0.05	0.08

Table S2. Baseline demographic characteristics of AVG and AVF groups before and after propensity score matching.

**Obese patients**	**Before Matching**			**After Matching**		
Characteristic Name: Demographics	AVG	AVF	Std. Diff.	AVG	AVF	Std. Diff.
Female	0.49	0.49	0.006	0.49	0.49	0.005
Male	0.49	0.48	0.014	0.48	0.48	0.005
White	0.42	0.45	0.048	0.44	0.45	0.004
Black or African American	0.41	0.39	0.034	0.40	0.40	0.012
Asian	0.02	0.02	0.009	0.02	0.02	0.006
**Normal patients**	**Before Matching**			**After Matching**		
Characteristic Name: Demographics	AVG	AVF	Std. Diff.	AVG	AVF	Std. Diff.
Female	0.57	0.55	0.04	0.56	0.56	0.00
Male	0.40	0.41	0.02	0.41	0.41	0.00
White	0.38	0.40	0.03	0.40	0.40	0.00
Black or African American	0.38	0.37	0.01	0.38	0.37	0.01
Asian	0.08	0.07	0.06	0.06	0.07	0.03

Table S3. Baseline diagnostic characteristics of AVG and AVF groups before and after propensity score matching.

**Obese patients**	**Before Matching**			**After Matching**		
Characteristic Name: Diagnoses	AVG	AVF	Std. Diff.	AVG	AVF	Std. Diff.
Hypertensive diseases	0.75	0.79	0.08	0.78	0.79	0.00
Diabetes mellitus	0.56	0.59	0.06	0.59	0.59	0.00
Ischemic heart diseases	0.31	0.32	0.00	0.31	0.32	0.01
Cerebrovascular diseases	0.11	0.11	0.01	0.11	0.11	0.00
Antiarrhythmics	0.52	0.59	0.13	0.58	0.58	0.00
Beta Blockers/Related	0.55	0.56	0.01	0.56	0.56	0.00
Blood Glucose Regulation Agents	0.52	0.52	0.01	0.52	0.53	0.00
Antilipemic Agents	0.40	0.41	0.02	0.41	0.41	0.00
Calcium Channel Blockers	0.39	0.38	0.01	0.38	0.38	0.00
Diuretics	0.40	0.38	0.04	0.38	0.38	0.00
Ace Inhibitors	0.17	0.15	0.04	0.16	0.15	0.01
Angiotensin ii Inhibitor	0.11	0.11	0.00	0.11	0.11	0.00
Alpha Blockers/Related	0.09	0.08	0.02	0.09	0.09	0.00

**Normal patients**	**Before Matching**			**After Matching**		
Characteristic Name: Diagnoses	AVG	AVF	Std. Diff.	AVG	AVF	Std. Diff.
Hypertensive diseases	0.76	0.80	0.08	0.79	0.80	0.01
Diabetes mellitus	0.44	0.46	0.03	0.46	0.46	0.00
Ischemic heart diseases	0.32	0.33	0.01	0.32	0.33	0.02
Cerebrovascular diseases	0.13	0.14	0.03	0.14	0.14	0.00
Antiarrhythmics	0.57	0.59	0.03	0.58	0.59	0.01
Beta Blockers/Related	0.51	0.57	0.12	0.55	0.56	0.02
Blood Glucose Regulation Agents	0.50	0.48	0.03	0.48	0.49	0.01
Antilipemic Agents	0.45	0.44	0.02	0.43	0.44	0.02
Calcium Channel Blockers	0.36	0.37	0.01	0.36	0.37	0.01
Diuretics	0.35	0.34	0.03	0.33	0.34	0.01
Ace Inhibitors	0.21	0.21	0.00	0.21	0.21	0.01
Angiotensin ii Inhibitor	0.12	0.11	0.02	0.11	0.11	0.01
Alpha Blockers/Related	0.10	0.10	0.01	0.10	0.10	0.00

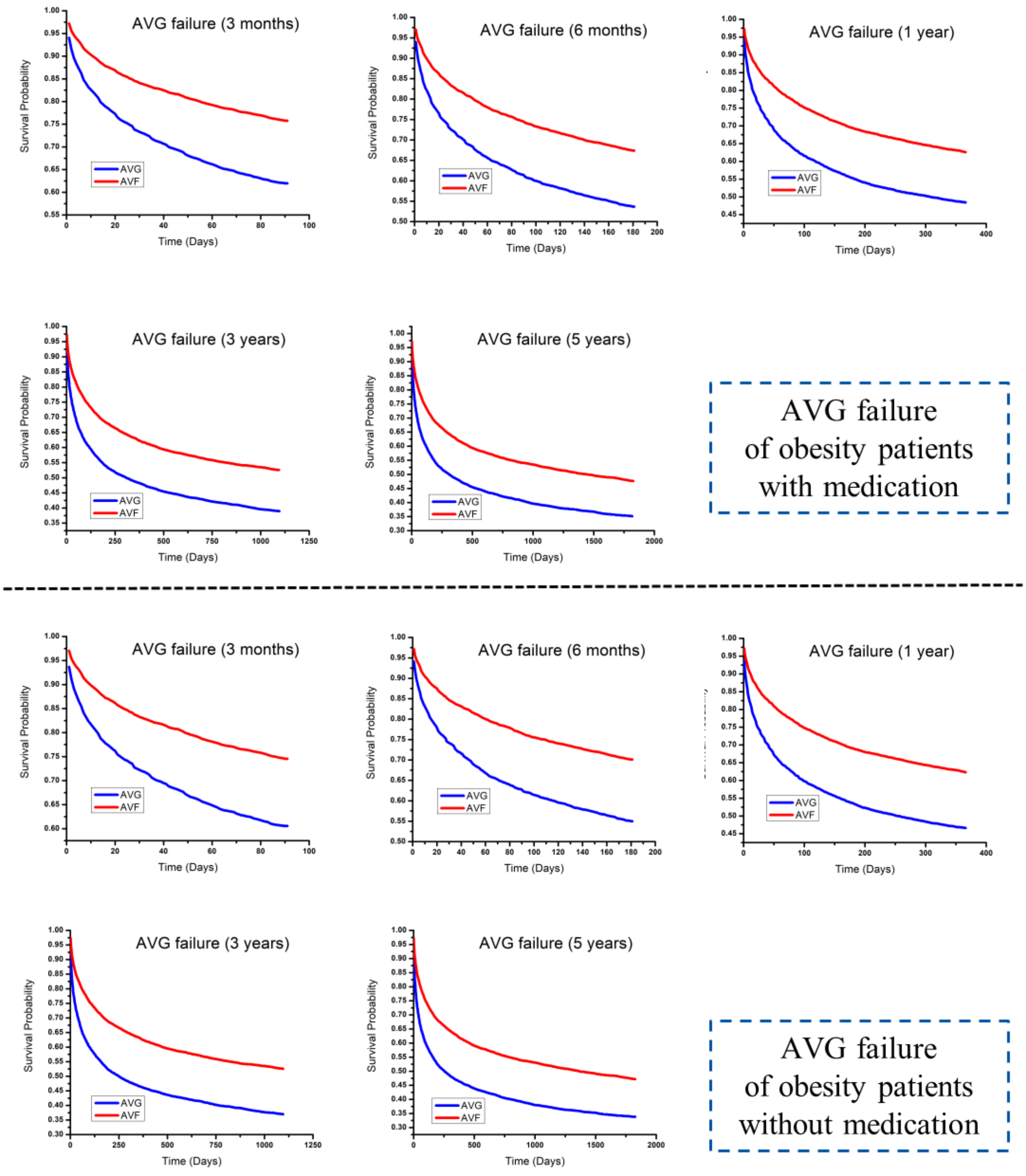

Figure S1. Kaplan-Meier survival curves of obesity patients for the 3-month to 5-year association of AVG with or without medication.

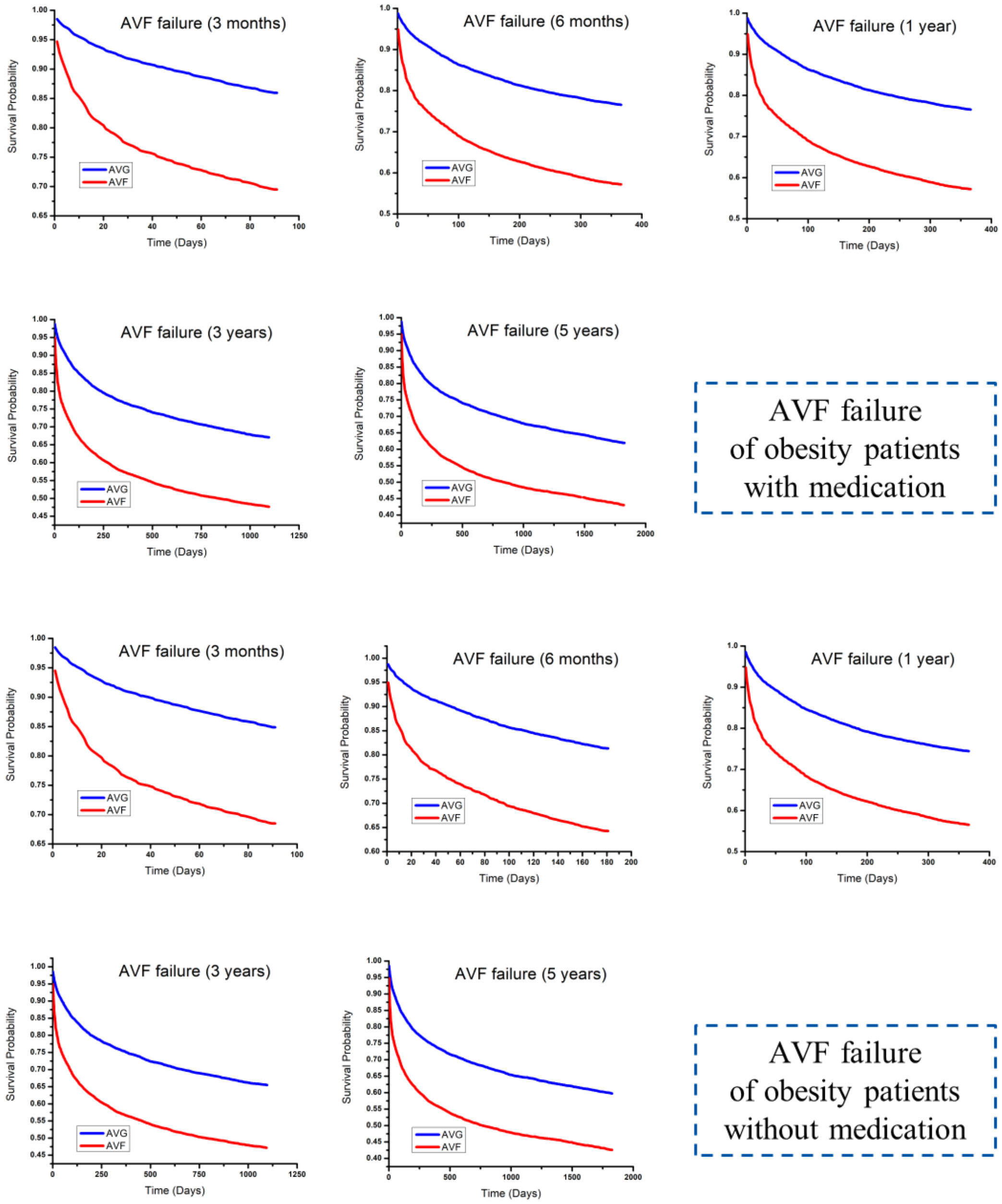

Figure S2. Kaplan-Meier survival curves of obesity patients for the 3-month to 5-year association of AVF with or without medication.

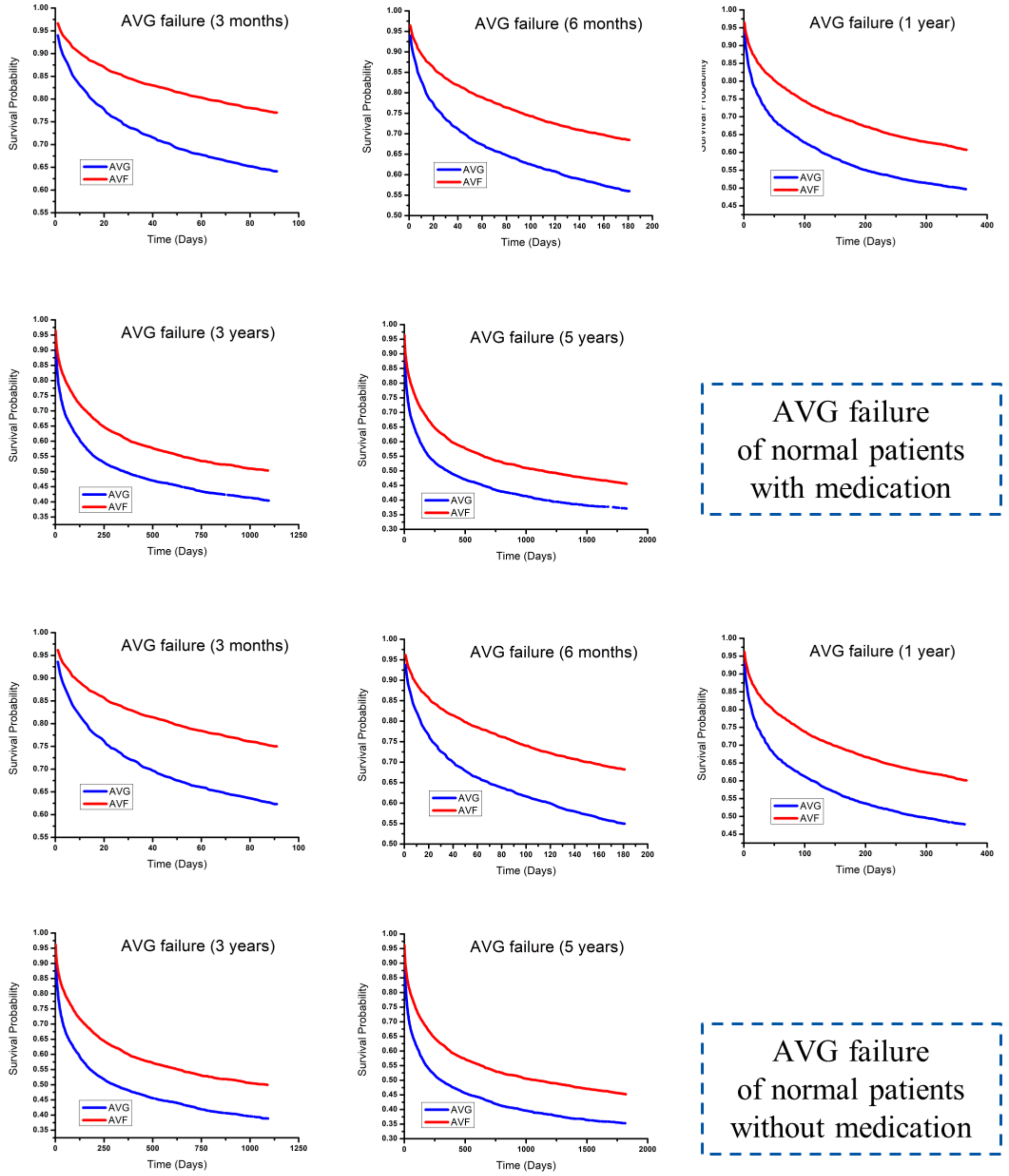

Figure S3. Kaplan-Meier survival curves of normal patients for the 3-month to 5-year association of AVG with or without medication.

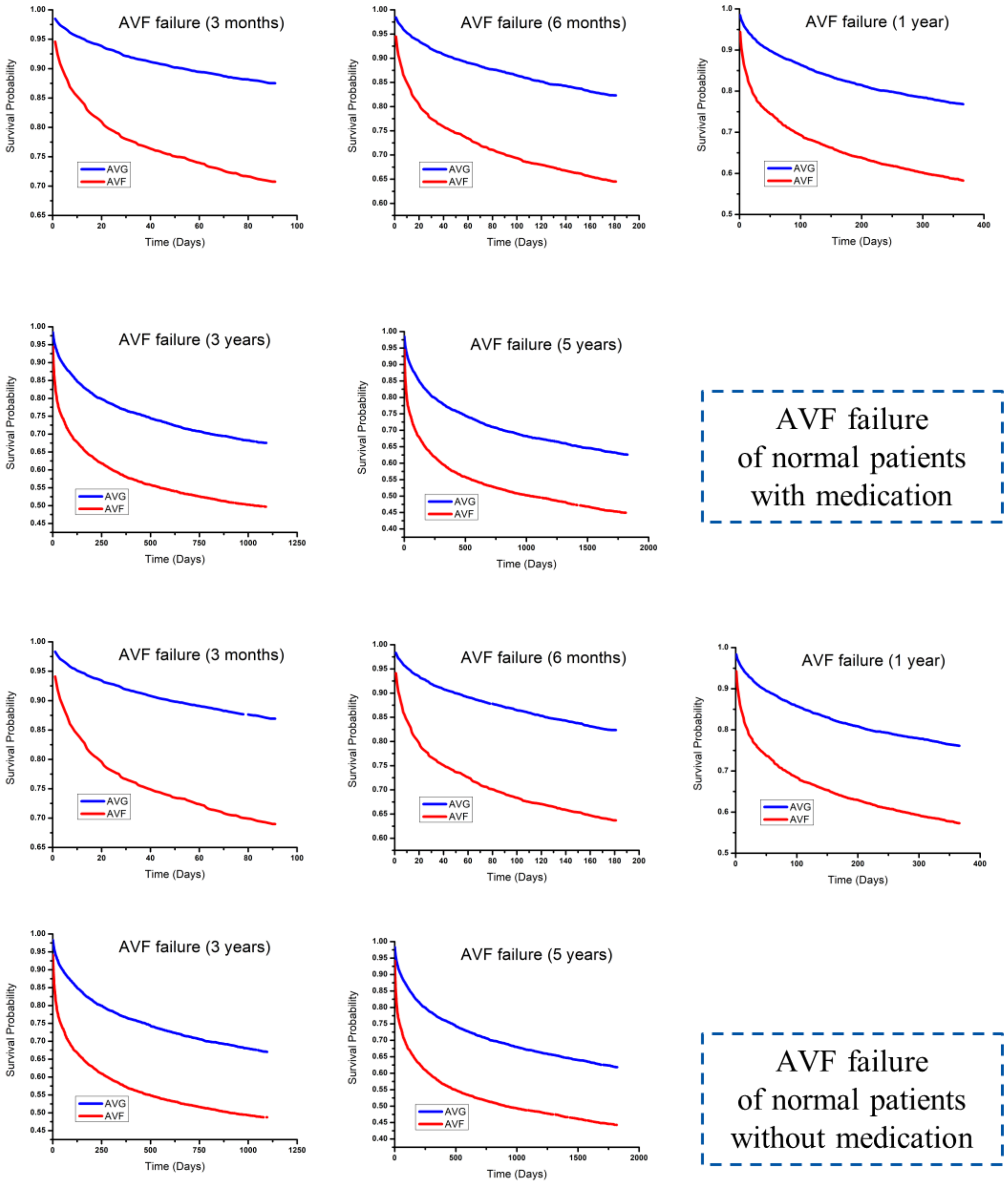

Figure S4. Kaplan-Meier survival curves of normal patients for the 3-month to 5-year association of AVF with or without medication.
